# Impact of epidermal growth factor receptor (*EGFR*) activating mutations and their targeted treatment in the prognosis of stage IV non-small cell lung cancer (NSCLC) patients harboring liver metastasis

**DOI:** 10.1186/s12967-015-0622-x

**Published:** 2015-08-07

**Authors:** Eduardo Castañón, Christian Rolfo, David Viñal, Inés López, Juan P Fusco, Marta Santisteban, Patricia Martin, Leire Zubiri, José I Echeveste, Ignacio Gil-Bazo

**Affiliations:** Department of Oncology, Clínica Universidad de Navarra, Avenida Pío XII, 36, 31008 Pamplona, Spain; Program in Solid Tumors and Biomarkers, Center for Applied Medical Research (CIMA), Pamplona, Spain; Department of Oncology, Antwerp University Hospital UZA, Edegem, Belgium; Department of Pathology, Clínica Universidad de Navarra, Pamplona, Spain

**Keywords:** Liver metastasis, Non-small cell lung cancer (NSCLC), Epidermal growth factor receptor (EGFR), EGFR tyrosin-kinase inhibitors, Prognosis

## Abstract

**Objectives:**

Liver metastases appear in 20–30% of patients diagnosed with non-small cell lung cancer (NSCLC) and represent a poor prognosis feature of NSCLC and a possibly more treatment-resistant condition. Potential clinical outcome differences in NSCLC patients with liver metastases harboring molecular alterations in *EGFR*, *KRAS* and *EML4*-*ALK* genes are still to be determined. This study aims to evaluate the incidence of liver metastasis in a single population and look for potential correlations between *EGFR* mutations, liver infiltration and clinical outcomes.

**Methods:**

A total of 236 consecutive stage IV NSCLC patients treated at the Clínica Universidad de Navarra were analyzed.

**Results:**

At onset, liver metastases were present in 16.9% of patients conferring them a shorter overall survival (OS) compared to those with different metastatic locations excluding liver infiltration (10 vs. 21 months; p = 0.001). Patients with *EGFR* wild-type tumors receiving standard chemotherapy and showing no liver involvement presented a superior median OS compared to those with liver metastases (23 vs. 13 months; p = 0.001). Conversely, patients with *EGFR*-mutated tumors treated with EGFR tyrosin-kinase inhibitors (TKI’s) presented no significant differences in OS regardless of liver involvement (median OS not reached vs. 25 months; p = 0.81).

**Conclusion:**

Overall, liver metastases at onset negatively impact OS of NSCLC patients. EGFR TKIs however, may reverse the effects of an initial negative prognosis of liver metastasis in first-line treatment of *EGFR* mutated NSCLC patients.

**Electronic supplementary material:**

The online version of this article (doi:10.1186/s12967-015-0622-x) contains supplementary material, which is available to authorized users.

## Background

More than 1,820,000 new cases of lung cancer were diagnosed in 2012 worldwide, leading to more than 1,500,000 annual deaths [1]. More than 50% of the patients diagnosed with non-small cell lung cancer (NSCLC) are expected to present advanced disease (stage III and IV) at onset associated with an overall survival (OS) barely exceeding 10–12 months from treatment initiation [2].

Although the most common site of metastases in stage IV NSCLC subjects is still to be determined [3], the organs most commonly colonized by primary non-small cell lung tumors are brain, bone, lung, adrenal glands, and liver.

It has been also postulated that those different metastatic locations may confer a diverse prognosis [3] and/or likelihood of disease response to the treatment [4].

In light of recent research on biological targets and the progressive development of new tailored therapies in NSCLC patients, there is a need to reassess the prognostic/predictive implications of different metastatic locations. Additionally, the recent development of new local (mainly ionizing radiation-based) therapies has contributed to the improved survival of patients with brain metastases [5], a metastatic location that has been traditionally known to entail a particularly poor prognosis [6].

Liver involvement appears in a range of 20–30% of NSCLC patients during the disease course conferring a significantly poorer prognosis to NSCLC at any stage of the disease since no local consolidation therapies are commonly used on a routine clinical practice [7].

Nevertheless, the impact different tumor histology and molecular characteristics may have on the spread of the disease to the liver is unknown. The current study aims to analyze the last consecutive stage IV NSCLC patients treated at our institution, paying special attention to the prevalence and outcome implications of liver metastases and correlated to the *EGFR* mutation status.

## Patients and methods

### Patients’ clinical and molecular characteristics

This study was designed as a retrospective analysis of a single cohort, reviewing and including the medical records of 236 histologically-confirmed stage IV NSCLC consecutive patients starting systemic antineoplastic treatment from 2006 to 2014. All patients signed informed consent before undergoing any diagnostic procedure leading to the obtention of tumor samples, or follow up imaging studies that required so. The only patient selection criterion applied was the availability of basic clinical information about the tumor histology confirmation, treatment administrated and at least 6 months follow-up.

All available characteristics including tumor histology and molecular features, the number of metastatic locations and organs affected were collected from patients’ medical charts. Overall, 236 consecutive NSCLC patients diagnosed with metastatic disease at the time of presentation were reviewed and included in the analysis. Main patients’ characteristics including their tumors’ histological patterns are summarized in Additional file 1: Table S1.

The molecular analysis performed on tumor samples included the assessment of Epidermal Growth Factor Receptor (*EGFR*) mutations in exons 18, 19, 20 and 21 and the assessment of Kristen Rat Sarcoma (*KRAS*) gene mutations in codons 12 and 13.

In brief, after the sample was fixed in alcohol and stained by Papanicolau stain, DNA was extracted and amplified via a PCR technique that uses *EGFR* gene exons 18, 19, 20 and 21 specific primers. ABI PRISM^®^ 310 Genetic Analyzer equipment was used for the analysis of the sequencing reactions with both forward and reverse primers. Following the same process used for *EGFR* analysis, PCR was used for DNA amplification, using *KRAS* gene exon 2 primer. ABI PRISM^®^ 310 Genetic Analyzer equipment was also used for the analysis of the sequencing reactions with both forward and reverse primers.

Smoking habits were also collected in accordance with the National Health Interview Survey (NHIS) for standard smoking definitions [8].

The protocol was approved by the Research Ethics Committee of the University of Navarra.

### Outcome measures

Patients were regularly followed during treatment for clinical and radiological response assessment. Computerized tomography (CT) scans were performed every 6 weeks as per institutional standard protocol.

The best radiological response to treatment was reviewed according to Response Evaluation Criteria In Solid Tumors (RECIST v1.1).

OS was calculated and compared between different groups. OS was calculated from the date of diagnosis to the date of death, lost to follow up, or last contact with the patient.

### Statistical analysis

To analyze the quantitative variables within the study population, a comparison was made using a Shapiro–Wilk test between the distribution of patients showing liver metastasis and those without liver involvement. Depending on the results of this test, either an unpaired 2-tailed Student’s t test or Mann Whitney test for parametric and non-parametric distribution, were used. A Chi2 test was also used to analyze the qualitative variables. OS was analyzed using the Kaplan–Meier (KM) method. A Log rank test or Taron Ware test was used to evaluate the differences among survival curves according to the curves’ distribution. We performed a multivariate regression model using Cox Regression method. A p value of <0.05 was considered statistically significant. Statistical analyses were conducted with the IBM SPPS statistical software package (v15.0 SPSS Inc. Chicago, IL).

## Results

### Molecular analysis

An *EGFR* mutational study was available in 73.7% of the patients (n = 174). *KRAS* was also studied in 44% of the population (n = 104).

Molecular findings are reflected in Additional file 2: Table S2, including the specification of the mutations detected. EGFR mutational status according to the pattern of metastasis at onset and during disease curse is summarized in Additional file 3: Table S3.

### Pattern of metastases

The most prevalent site of metastasis at onset corresponded to bone (94/236; 39.8%) followed by brain (75/236; 31.8%), adrenal glands (44/236; 18.6%), liver (40/236; 16.9%), bilateral lung lesions (25/236; 10.6%), pleural implants (19/236; 8.1%) and skin metastases (10/236; 4.2%). The final metastatic localization developed during treatment and follow-up was as follows, bone (49.2%), brain (42.8%), liver (39%), adrenal glands (28.4%), bilateral lung involvement (18.2%), pleural implants (11%) and skin metastases (7.2%).

### Liver involvement implications on patients’ epidemiology

We divided our population in two groups, whether liver involvement was present or not. Both groups of patients were compared in order to detect potentially different clinical characteristics among those patients harboring liver metastases and those with NSCLC who had metastasized to different organs other than the liver. No significant differences in gender, age, ECOG performance status (PS) or smoking history were found to be correlated to liver involvement (Chi squared test, T test and Mann Witney test were used for categorical and quantitative variables; p = 0.62, p = 0.05; p = 0.35; p = 0.23, respectively). The total number of chemotherapy lines received was slightly higher among subjects without liver dissemination compared to those showing liver involvement (mean 2.41, 95% CI 2.1–2.7 vs. 1.84, CI 1.6–2.03, respectively; T test result: p = 0.001).

A higher proportion of tumors with adenocarcinoma histology among patients who were not exhibiting liver metastasis was observed (83.3 vs. 52.2%; Chi square test: p = 0.001), whereas large-cell carcinoma seemed to be significantly more frequent among patients with liver involvement (16.3 vs. 6.25%; Chi square test: p = 0.01). Patients with squamous cell histology tumors were slightly higher prevalent in the group with liver metastases compared to those without liver spread disease (16.3 vs. 9.7%; Chi square test: p = 0.09) although no statistical differences were observed.

Regardless of liver involvement, *EGFR* and *KRAS* status were equally balanced among patients, (Chi square test: p = 0.83 and p = 0.78, respectively).

As expected, the use of first-line EGFR TKIs was significantly related to the *EGFR* mutation status. In fact, while the first-line EGFR TKIs use among *EGFR* mutated patients was 56.7% (17/30), it was 2.1% (3/144) among *EGFR* wild type patients (Chi square test: p = 0.001). The use of EGFR TKIs as a second or subsequent line in patients with *EGFR* mutated tumors was 42.8% (12/30) compared to 19.8% (26/144) in subjects with *EGFR* wild type tumors (Chi square test: p = 0.008). However, their use did no differ from patients without liver metastases compared to those with secondary liver involvement (12.6 vs. 9.1%; Chi square test: p = 0.49).

### Survival impact

**Fig. 1:**
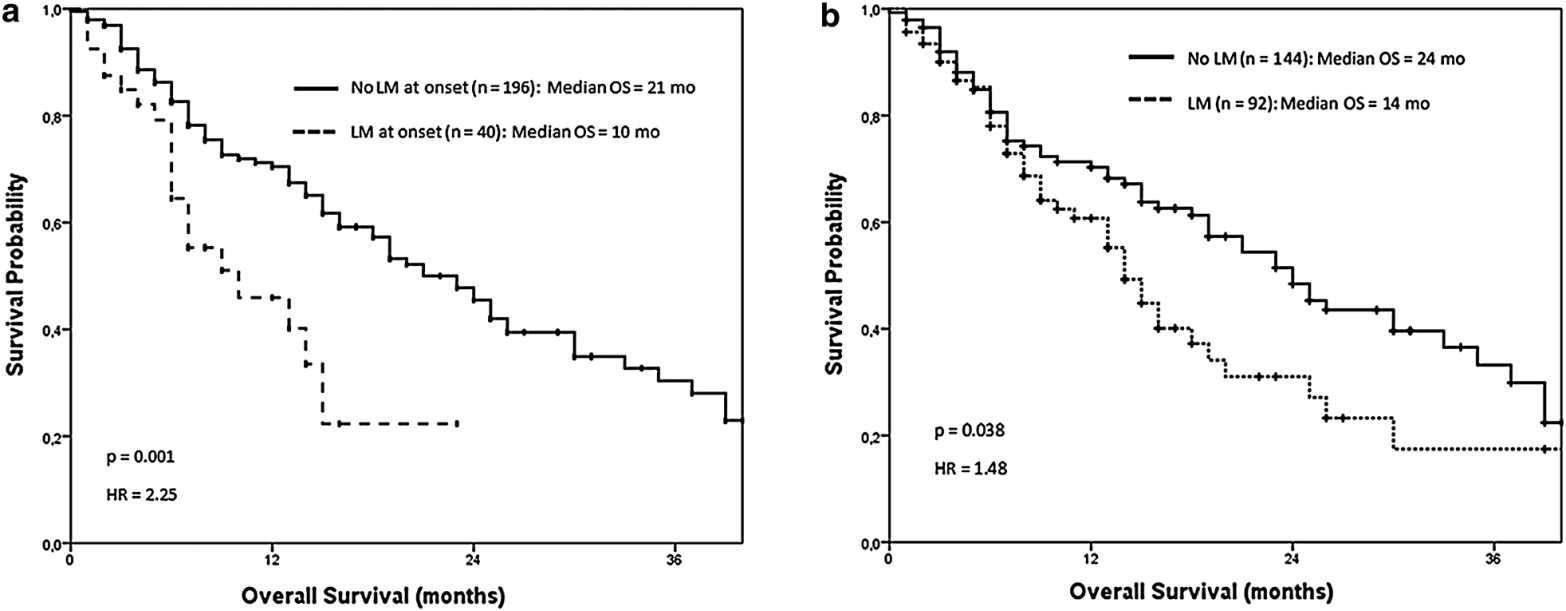
OS depending on the presence of LM at onset or during disease curse. **a** At onset, LM presence seems to be a poor prognosis factor detecting an OS of 10 months when LM are present (n = 40) compared to 21 months when no liver metastases are diagnosed at the moment of stage IV NSCLC diagnosis (n = 196). **b** A better overall survival is achieved in stage IV NSCLC patients who never present liver involvement (n = 144) compared to those patients in whom LM are present during the course of the disease (n = 86).

As expected, a significant OS benefit was observed for patients with stage IV NSCLC showing no liver involvement at onset when compared to those exhibiting liver infiltration at the time of stage IV diagnosis [KM survival: 21 months (95% CI 16.9–25.1) vs. 10 months (95% CI 2.8–17.2); p = 0.001], (Fig. 1a). When taken into account the presence of LM at any time-point during the entire disease process, we found a shorter OS [14 months (95% CI 11.6–16.4)] in patients with liver disease, when compared to those patients in which liver was never affected [24 months (95% CI 18.6–29.3), p = 0.038], (Fig. 1b).

**Fig. 2:**
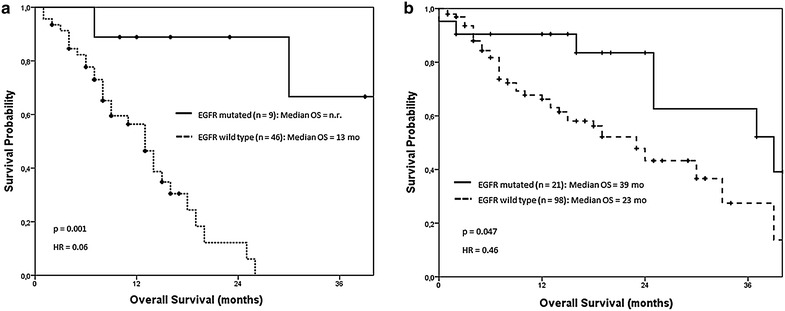
OS depending on the *EGFR* status stratified by the presence of LM. **a** A subanalysis was performed selecting those patients with liver involvement. We observed a significant difference between those patients harboring *EGFR* mutations (n = 9) compared to those showing wild-type *EGFR* (n = 46). **b** When selecting patients with no liver involvement, we also observed a better outcome for those harboring *EGFR* mutation (n = 21) compared to those with *EGFR* wild type NSCLC (n = 98).

Additionally, a subanalysis was performed in order to study the impact of *EGFR* mutation status and the treatment with EGFR TKIs on the OS according to the presence or absence of liver involvement. Among patients with liver involvement, those with *EGFR* mutated tumors experienced a significantly superior OS compared to subjects with *EGFR* wild-type neoplasms [median OS not reached (95% CI not reached (N.R.)] vs. 13 months (95% CI 10.2–15.7), respectively; (HR = 0.06; p = 0.001) (Fig. 2a). Similarly, patients without liver involvement and *EGFR* mutated tumors showed a superior OS compared to those with *EGFR* wild type NSCLC [39 months (95% CI 20.2–57.8) vs. 23 months (95% CI 17.2–28.8); p = 0.047], (Fig. 2b).

**Fig. 3:**
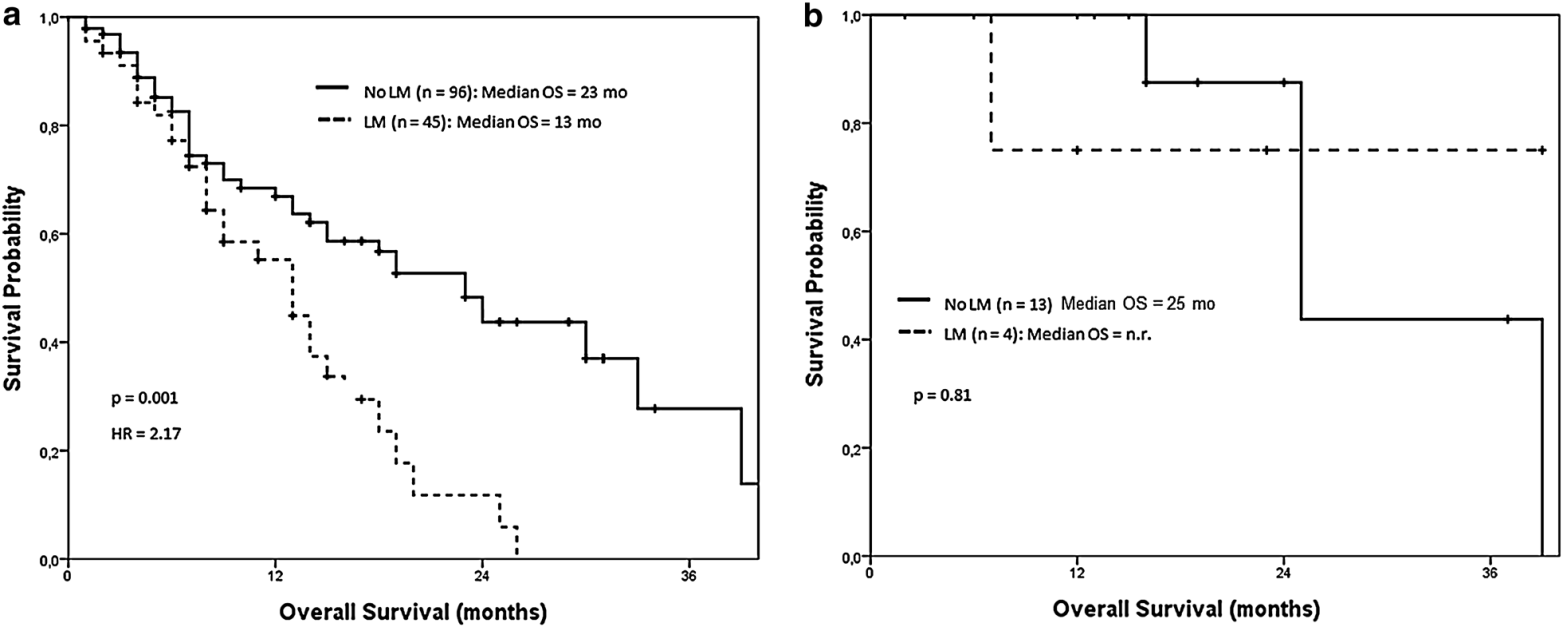
OS in patients with LM depending on *EGFR* status. **a** *EGFR* wild-type NSCLC patients who received standard first-line chemotherapy presented a clear benefit in terms of OS when liver was not involved (n = 96) compared to those patients with LM (n = 45). **b** No differences were observed in *EGFR* mutated population receiving first line TKI in terms of OS when comparing patients with no LM (n = 13) with patients with liver involvement (n = 4).

Subsequently, we analyzed the impact on the OS of the EGFR TKIs treatment in those patients with *EFGR* mutant tumors receiving first-line targeted therapy, according to the presence or absence of secondary liver involvement. On the one hand, when the OS analysis was restricted to *EGFR* wild-type NSCLC patients who received standard first-line chemotherapy, a clear benefit in OS was observed in favor of individuals without liver involvement compared to those patients with liver disease [23 months (95% CI 13.1–32.9) vs. 13 months (95% CI 8.1–17.9), respectively; p = 0.001]. In fact, those subjects showing liver involvement presented a 117% higher risk of death than patients with no liver involvement (HR = 2.17), (Fig. 3a). Interestingly enough and in contrast with our previous results, liver involvement lost its prognostic impact among patients with *EGFR* mutated tumors receiving first-line EGFR TKIs therapy. Thus, a non-statistically significant benefit in OS in subjects without liver metastases compared to those with liver involvement was observed [median OS not reached (95% CI N.R.) vs. 25 months (95% CI 15.8–34.1); p = 0.81], (Fig. 3b).

Finally, a Cox regression model was performed for assessing prognostic factors in terms of OS for the population in which *EGFR* mutation status had been studied (n = 174). In the univariate analysis, nodal involvement (HR = 1.29, p = 0.02), *EGFR* mutation (HR = 0.31, p = 0.01), use of EGFR TKIs after progression (HR = 0.4, p = 0.03), liver metastases at onset localized in liver (HR = 2.25, p = 0.001), bone (HR = 2.13, p = 0.001), adrenal glands (HR = 1.75, p = 0.03) and skin (HR = 4.67, p = 0.03) constituted factors related to OS. Also, the appearance of metastases in the liver (HR = 1.48, p = 0.04) and in the bone (HR = 1.93, p = 0.01) during the disease curse were also related to a poorer outcome (Additional file 4: Table S4). After introducing these variables in a multivariate analysis, metastatic localization, including liver involvement, lost its prognostic value when adjusted by *EGFR* mutations and TKIs use. In fact, the presence of *EGFR* mutations (HR = 0.24, p = 0.001) and the use of EGFR TKIs after progression to chemotherapy (HR = 0.44, p = 0.03), were independently related to a better survival in stage IV NSCLC, (Additional file 5: Tables S5).

## Discussion

It is known that the development of liver metastasis confers a poor prognosis to NSCLC patients, since no local treatments are still widely used to specifically target liver involvement in unselected patients [9, 10].

The first interesting finding in our study is that although liver involvement was present in only 16.3% of the patients at onset, the liver became the third most frequent site of metastasis during the course of the disease, closely following bone and brain locations and in accordance with previous reports [3].

More interestingly, we confirm that subjects with liver infiltration are among those who significantly obtain less clinical benefit from chemotherapy administration. Therefore, liver involvement seems to be a poor prognostic feature with a significantly reduced survival expectation compared to other patients with metastatic disease who never develop liver metastasis, as previously reported [4]. Moreover, brain metastasis seemed to provide even a better prognosis to the rest of the patients compared to liver involvement, most probably because in our clinical cohort all patients with brain metastasis were locally treated with either whole brain irradiation or stereotactic body radiation therapy.

Furthermore, we found that the tumor genotype seemed to correlate with the pattern of metastasis observed. A previous study by Doebele et al. [11] showed that NSCLC patients with *EGFR* mutations were significantly predisposed to liver metastasis compared to the triple negative cohort. In contrast, in our series *EGFR* mutant patients did not seem to be more prone to developing liver metastasis compared to *EGFR* wild type patients. Potential different characteristics in our population, in which for example *ALK* rearrangement was not studied, may explain the differences detected compared to the study by Doebele el al.

It has been extensively demonstrated that NSCLC patients with *EGFR* mutated tumors clinically benefit from receiving a first-line treatment with an EGFR TKI, such as erlotinib [12], gefitinib [13] or afatinib [14] reaching a median progression free survival of around 10 months and an OS superior to 22 months in most of the published clinical trials [13–15]. However, in those clinical trials no specific subanalysis showing the clinical outcome of patients with liver metastasis has been reported.

Therefore, in the present study we aimed to investigate whether the molecular tumor subtype influenced the clinical outcome of NSCLC patients with liver involvement, and more importantly, by the targeted therapy received.

For the first time in the literature, we found that the OS of subjects with liver involvement does not significantly differ from that of those with different metastatic locations other than the liver, when considering the subpopulation of patients with *EGFR*-mutated tumors treated with a first-line EGFR TKI. In contrast to what it is observed in *EGFR* wild-type patients receiving standard chemotherapy. This finding would suggest that the sensitivity to EGFR targeted therapies in *EGFR* mutant NSCLC patients is not compromised in any way by the specific location of the metastasis. On the other hand, this observation could be well explained by the fact that chemotherapy induces lower response rates and requires more time to produce a clinically relevant response in EGFR wild type patients compared to EGFR TKIs in subjects with mutated tumors. In that case, liver infiltration would cause a faster clinical deterioration and a rapid worsening of the patient’s performance status with a subsequent earlier discontinuation of active treatment. In fact, the total number of chemotherapy lines received was significantly higher among subjects without liver dissemination compared to those showing liver involvement. In contrast, the early clinical and radiological response commonly seen among NSCLC patients with tumors harboring *EGFR* activating mutations who receive EGFR TKIs would contribute to a rapid improvement in the patient’s clinical condition and PS, allowing those patients to continue treatment for longer periods benefiting more extensively from therapy.

Some early clinical data have shown that EFGR TKIs gefitinib and erlotinib are mainly metabolized in the liver. Liver dysfunction may contribute to an overexposure to the drug [16–18]. Therefore, it could be hypothesized that the overexposure to the drug may lead to a potentially higher efficacy of EGFR TKIs among patients with liver dysfunction resulting from liver metastases.

The retrospective nature of this study and some lacking data are potential limitations for the present study. More specifically, in the subgroup analysis of *EGFR* mutated tumors in patients receiving first-line EGFR TKIs with regards to liver involvement. Nevertheless, the total number of patients analyzed and the results obtained therein warrant a thorough analysis of previous prospective trials in order to identify potential differences in survival that may result from the metastatic locations and the molecular profile of the tumors.

## Conclusions

Subjects with NSCLC and liver infiltration present an overall poorer prognosis obtaining less clinical benefit from chemotherapy administration. Conversely, the OS of subjects with liver involvement did not significantly differ from those with other metastatic locations when considering the subpopulation of patients with *EGFR* mutations harboring tumors treated with a first-line EGFR TKI.

## Additional files

Additional file 1:
**Table S1.** Patients’ features at the moment of enrollment in the study depending on the presence or absence of liver metastases. Additional file 2:
**Table S2.** Molecular findings in our clinical series. *EGFR* (Epidermal Growth Factor Receptor), *KRAS* (Kristen Rat sarcoma). Additional file 3:
**Table S3.** Metastatic location depending on *EGFR* (Epidermal Growth Factor Receptor) status. Additional file 4:
**Table S4.** Univariate Regression Model for predicting OS (overall survival) in our clinical series. Additional file 5:
**Table S5.** Multivariate regression model for predicting OS (overall survival) in our clinical series.

## Abbreviations

CI: confidence interval
CT: computerized tomography
DNA: deoxyribonucleic acid
ECOG: Eastern Cooperative Oncology Group
EGFR: epidermal growth factor receptor
FISH: fluorescence in situ hybridization
HR: hazard ratio
N.R.: not reached
NHIS: National Health Interview Survey
NOS: not otherwise specified
NSCLC: non-small cell lung cancer
OS: overall survival
PFS: progression free survival
PCR: polymerase chain reaction
PS: performance status
RECIST: response evaluation criteria in solid tumors
KRAS: Kristen rat sarcoma (GTPase)
SBRT: stereotactic body radiation therapy
TKI: tyrosine kinase inhibitor
WBI: whole brain irradiation
