# Supplementary material for: Impact of epidermal growth factor receptor (EGFR) activating mutations and their targeted treatment in the prognosis of stage IV non-small cell lung cancer (NSCLC) patients harboring liver metastasis
Source: J Transl Med. 2015 Aug 7;13:257. doi: 10.1186/s12967-015-0622-x (PMC4528698; doi:10.1186/s12967-015-0622-x)
Supplement: Additional file 1: — Table S1. Patients’ features at the moment of enrollment in the study depending on the presence or absence of liver metastases. [file 12967_2015_622_MOESM1_ESM.pdf]

Characteristics	No liver involvement at onset	Liver metastasis at onset	p value
**Sex**			
Male	133 (67.9%)	33 (82.5%)	0.08
Female	66 (32.1%)	7 (17.5%)	
**T**			0.4
Tx	11 (5.6%)	0 (0%)	
T1	30 (15.3%)	5 (12.5%)	
T2	64 (32.7%)	10 (25%)	
T3	28 (14.3%)	7 (17.5%)	
T4	63 (32.1%)	18 (45%)	
**N**			0.3
Nx	4 (2 %)		
N0	33 (16.8%)	6 (15%)	
N1	10 (5.1%)	3 (7.5%)	
N2	81 (41.3%)	11 (27.5%)	
N3	68 (34.7%)	20 (50%)	
**M1**	196	40	-
**Bone metastases**	89 (45.4%)	27 (67.5%)	0.01
**Bone metastases at onset**	69 (35.2%)	25 (62.5%)	0.01
**Skin metastases**	13 (6.6%)	4 (10%)	0.5
**Skin metastases at onset**	7 (3.6%)	3 (7.5%)	0.3
**Adrenal metastases**	55 (28.1%)	12(30%)	0.8
**Adrenal metastases at onset**	33 (16.8%)	11 (27.5%)	0.1
**Brain metastases**	88 (44.9%)	13(32.5%)	0.1
**Brain metastases at onset**	66 (33.7%)	9 (22.5%)	0.2
**Smoker**			0.07
Non smoker	46 (23.5%)	10 (25%)	
Ex smoker	98 (50%)	15 (37.5%)	
Smoker	50 (25.5)	14 (35%)	
**EGFR**			0.06
Wild type	125 (63.8%)	19 (47.5%)	
Mutate	26 (13.3%)	4 (10%)	
Unknown	45 (23%)	17 (42.5%)	
**KRAS**			0.4
Wild type	71 (36.2%)	11 (27.5%)	
Mutated	19 (9.7%)	3 (7.5%)	
Unknown	106 (54.1%)	26 (65%)	
**Histology**			0.2
Adenocarcinoma	145 (74%)	23 (57.5%)	
Squamous cell	22 (11.2%)	7 (17.5%)	
Adenosquamous	2 (1%)	0 (0%)	
Giant cell	17 (8.7%)	7 (17.5%)	
Undifferentiated	10 (5.1%)	3 (7.5%)	
**Median ECOG (range)**	1 (0-2)	1 (0-2)	0.6
**Media Age (range)**	65.5 (47-89)	62 (35-84)	0.1
**Median Number of lines (range)**	2 (1-7)	2 (1-6)	0.8
