# Supplementary material for: Impact of epidermal growth factor receptor (EGFR) activating mutations and their targeted treatment in the prognosis of stage IV non-small cell lung cancer (NSCLC) patients harboring liver metastasis
Source: J Transl Med. 2015 Aug 7;13:257. doi: 10.1186/s12967-015-0622-x (PMC4528698; doi:10.1186/s12967-015-0622-x)
Supplement: Additional file 2: — Table S2. Molecular findings in our clinical series. EGFR (Epidermal Growth Factor Receptor), KRAS (Kristen Rat sarcoma). [file 12967_2015_622_MOESM2_ESM.pdf]

EGFR analyzed			
	Wild type		144 (82.7%)
	Mutated		30 (17.3%)
		Exon 19	18 (60%)
		Exon 21	8 (26.7%)
		Exon 18	1 (3.3%)
		Exon 20	1 (3.3%)
		Not available	2 (6.7%)
KRAS analyzed			
	Wild Type		82 (78.8%)
	Mutated		22 (21.2%)
		G12D	6 (27.3%)
		G12V	4 (18.2%)
		G12C	2 (9.1%)
		G13D	2 (9.1%)
		G12A	1 (4.5%)
		G12S	1 (4.5%)
		L19F	1 (4.5%)
		Not specified	5 (22.7%)
