# Supplementary material for: Impact of epidermal growth factor receptor (EGFR) activating mutations and their targeted treatment in the prognosis of stage IV non-small cell lung cancer (NSCLC) patients harboring liver metastasis
Source: J Transl Med. 2015 Aug 7;13:257. doi: 10.1186/s12967-015-0622-x (PMC4528698; doi:10.1186/s12967-015-0622-x)
Supplement: Additional file 3: — Table S3. Metastatic location depending on EGFR (Epidermal Growth Factor Receptor) status. [file 12967_2015_622_MOESM3_ESM.pdf]

Metastasis Localization	EGFR wild type (n = 144)	EGFR mutated (n = 29)	p value
Bone metastases at onset	58 (40.3%)	15 (51.7%)	0.9
Bone metastases during disease	73 (50.7%)	15 (51.7%)	0.9
Liver metastases at onset	19 (13.2%)	4 (13.8%)	0.9
Liver metastases during disease	46 (31.9%)	9 (31%)	0.8
Adrenal metastases at onset	26 (18.1%)	6 (20.7%)	0.7
Adrenal metastases during disease	38 (26.4%)	10 (34.5%)	0.3
Brain metastases at onset	46 (31.9%)	12 (41.4%)	0.3
Brain metastases during disease	38 (26.4%)	10 (34.5%)	0.7
Skin metastases at onset	3 (2.1%)	0 (0%)	0.9
Skin metastases during disease	9 (6.3%)	0 (0%)	0.4
Bilateral lung lesions at onset	22 (15.3%)	5 (17.2%)	0.8
Bilateral lung lesions during disease	23 (15.9%)	6 (20.7%)	0.6
