# Supplementary material for: Impact of epidermal growth factor receptor (EGFR) activating mutations and their targeted treatment in the prognosis of stage IV non-small cell lung cancer (NSCLC) patients harboring liver metastasis
Source: J Transl Med. 2015 Aug 7;13:257. doi: 10.1186/s12967-015-0622-x (PMC4528698; doi:10.1186/s12967-015-0622-x)
Supplement: Additional file 4: — Table S4. Univariate Regression Model for predicting OS (overall survival) in our clinical series. [file 12967_2015_622_MOESM4_ESM.pdf]

Variable	HR	p
ECOG	1.03	0.123
Sex	1.01	0.2
Age	0.79	0.31
Smoker status	0.9	0.99
T	1.03	0.74
N	1.29	0.02
EGFR	0.31	0.01
KRAS	1.02	0.95
Histology	1.01	0.95
Number of lines	0.94	0.22
TKIs (first line)	0.45	0.06
TKIs (after progression)	0.4	0.03
Liver metastases at onset	2.28	0.009
Liver metastases during disease	1.55	0.04
Bone metastases at onset	2.13	0.001
Bone metastases during disease	1.93	0.01
Brain metastases at onset	1.16	0.5
Brain metastases during disease	0.98	0.93
Skin metastases at onset	4.67	0.03
Skin metastases during disease	0.68	0.51
Adrenal metastases at onset	1.75	0.03
Adrenal metastases during disease	1.46	0.1
Bilateral lung lesions at onset	1.06	0.8
Bilateral lung lesions during disease	0.82	0.9
