# Supplementary material for: Impact of epidermal growth factor receptor (EGFR) activating mutations and their targeted treatment in the prognosis of stage IV non-small cell lung cancer (NSCLC) patients harboring liver metastasis
Source: J Transl Med. 2015 Aug 7;13:257. doi: 10.1186/s12967-015-0622-x (PMC4528698; doi:10.1186/s12967-015-0622-x)
Supplement: Additional file 5: — Table S5. Multivariate regression model for predicting OS (overall survival) in our clinical series. [file 12967_2015_622_MOESM5_ESM.pdf]

**Variable**	**HR**	**p**
Sex	1.28	0.32
Age	1	0.9
N	1.28	0.06
EGFR	0.24	0.001
TKIs (after progression)	0.44	0.03
Liver metastases at onset	1.5	0.28
Liver metastases during disease	1.28	0.43
Bone metastases at onset	1.6	0.22
Bone metastases during disease	1.19	0.64
Skin metastases at onset	2.2	0.31
Adrenal metastases at onset	1.37	0.29
